# Metabolomics: a tool for the diagnosis of GH deficiency and for monitoring GH replacement?

**DOI:** 10.1530/EC-14-0098

**Published:** 2014-10-29

**Authors:** Charlotte Höybye, Erik Wahlström, Petra Tollet-Egnell, Gunnar Norstedt

**Affiliations:** 1 Department of Endocrinology, Metabolism and Diabetology, Karolinska University Hospital, 171 76 Stockholm, Sweden; 2 Department of Molecular Medicine and Surgery, Karolinska Institute, Stockholm, Sweden

**Keywords:** metabolomics, GHD in adults, GH treatment

## Abstract

The diagnostic value of insulin-like growth factor 1 (IGF1) for GH deficiency (GHD) in adults is not optimal. Molecular profiling could be used for biomarker discovery. The aim of this pilot study was to compare the serum metabolome between GHD patients and healthy controls, and identification of potential markers for diagnosis and/or for individual GH dosing. A total of ten patients with GHD, median age of 55 years and BMI of 27 kg/m^2^, were compared with ten healthy age- and gender-matched controls. The serum metabolic profiles were generated using gas chromatography-coupled mass spectroscopy on fasting samples taken in the morning from the controls and at baseline and during 6 months of GH replacement in the patients with GHD. The difference in low-molecular weight compounds (LMC) distinguished the healthy controls from GHD patients. Among 285 measured metabolites, 13 were identified as being most important in differentiating GHD patients from controls. Of these, 11 could not be structurally annotated but many were classified as lipids. The difference in the LMC pattern persisted despite normalisation of IGF1 following GH replacement. GH replacement increased the levels of specific fatty acid compounds and decreased the levels of certain amino acids. No metabolite changed in response to GH treatment, to the same extent as IGF1. The measurement of 285 metabolites resulted in a unique pattern in GHD, but changes in the metabolite patterns during GH treatment were limited. The utility of metabolomics to find new markers in GHD and GH replacement remains to be further elucidated.

## Introduction

Metabolomics is an exciting new analytical field in systems biology, which uses techniques such as mass spectrometry (MS) and nuclear magnetic resonance (NMR) spectroscopy to define the pattern of low-molecular weight compounds (LMCs) present in cells, tissues or biofluids (the metabolome) (1, 2). The resulting molecular fingerprint is the downstream result of gene transcription, translation and post-translational protein modifications in a cell, tissue or whole organism in a particular physiological state. As metabolite profiles of, for example, human serum are regarded as an important indicator of physiological or pathological states, such profiles may provide a method of identifying biomarkers of disease and treatment efficacy (3).

Adult growth hormone deficiency (GHD) syndrome is a well-defined clinical entity, including abnormal body composition, poor quality of life, dyslipidaemia and increased cardiovascular risk and mortality (4, 5). GH has systematically been given to adults with GHD for approximately two decades and it is well-known that it has beneficial effects on body composition, blood lipids, exercise capacity and quality of life (4, 5).

The effects of GH on adults is mediated directly through its own receptor and indirectly through production in the liver or the periphery of insulin-like growth factor 1 (IGF1). IGF1 circulates bound to a number of binding proteins, of which six high-affinity proteins have been identified and fully characterised. IGF-binding protein 3 (IGFBP3) is quantitatively the most important binding protein, carrying around three-quarters of total IGF1. As diurnal secretion of GH is pulsatile, evaluation of GH secretion needs repeated blood sampling or stimulation tests (4, 5, 6). In contrast, the production of IGF1 is more constant during 24 h, but the level is influenced by many factors, including nutritional state, age and gender. As a consequence, IGF1 is not an optimal marker for the diagnosis of GHD, but is been widely used for monitoring GH dosing (4, 5). Other markers for GH (IGFBP3 and acid labile subunit (ALS)) have been investigated; however, they have not improved diagnostic sensitivity (5). Therefore, it is of interest not only to find new markers for the diagnosis of GHD but also markers to monitor individual responses to GH therapy.

Recently, urine metabolic profiles using NMR spectroscopy in a patient with GHD were reported (7). The authors found that the metabolic profile was different in the patient as compared with healthy controls, and changes in metabolic profile during GH treatment and after discontinuation of GH replacement was observed. The aims of this study were to detect the levels of serum metabolome in GHD patients and healthy subjects and to find GH-dependent metabolites that might serve as new markers for the diagnosis of GHD and/or individual GH dose titration.

## Subjects and methods

### Patients

The study included ten patients, four men and six women median age of 55 years (26–62 years), BMI 27 kg/m^2^, with verified severe GHD, defined by a GH peak response of <3 μg/l after insulin hypoglycaemia and arginine stimulation test. All patients had three to four additional pituitary insufficiencies and had been receiving a stable conventional replacement therapy with thyroxin, hydrocortisone, sex steroids, and vasopressin for at least 2 years. The characteristics of the patients are given in Table 1. All had adult-onset GHD, and had not previously been treated with GH. None of the patients had diabetes.

### Healthy controls

At baseline, the patients with GHD were compared with ten age- and gender-matched healthy individuals. The metabolic and anthropometric characteristics of the controls are given in Table 2.

### Experimental protocol

The patients with GHD were administered one s.c. injection of GH at bedtime. An initial total daily GH dose of 0.10 mg was injected for a month, thereafter the doses were individually titrated to IGF1 levels in healthy subjects. The samples of patients were evaluated at baseline and after 1 and 6 months of GH treatment. The metabolic investigations were carried out at 0800 h after an overnight fast and there was no life style intervention. The baseline values of GHD patients were compared with the ten healthy controls.

The committee for medical ethics at the Karolinska Institute approved the study, which was carried out in accordance with the Declaration of Helsinki. All participants gave their consent to participate in the study before sample collection and examinations.

### Anthropometric methods

Anthropometric measurements were carried out at baseline in control and in the GHD adults at baseline and after 6 months of GH replacement.

Physical examination included measurements of height and weight. BMI was calculated as weight divided by the square of height in meters, kg/m^2^. Waist circumferences were measured in the standing position, measured halfway between the costal edge and iliac crest.

### Analysis

Fasting anti-coagulated blood was collected from the controls and the patients with GHD. Serum samples were stored at −70 °C until analysis.

### Assays

Blood glucose was determined by the glucose oxidase method using a standard glucose analyser (YSI, Inc., Yellow Springs, OH, USA). HbA1c concentration was analysed using the Mono-S method, which gives values approximately 1.1 percent-units lower than the DCCT standard.

Serum cholesterol and triglycerides (TG) were measured by colorimetric methods (Vitos 900) and HDL by direct calorimetry (Hitachi 911). LDL-cholesterol concentration was calculated according to Friedewald's formula (8).

IGF1 level was determined in serum by RIA (9). Normal range of IGF1 was established from 448 healthy subjects aged 20–96 years (10).

### Serum metabolites

LMCs from the serum samples were extracted and analysed by gas chromatography-coupled mass spectroscopy (GC/MS) (Pegasus III time-of-flight mass spectrometer, GC/TOFMS (Leco Corp., St Joseph, MI, USA)) according to a method developed previously (11).

All non-processed MS-files from the metabolic analysis were exported from the ChromaTOF software in NetCDF format to MATLAB software 7.0 (Mathworks, Natick, MA, USA), where all data on pre-treatment procedures, such as baseline correction chromatogram alignment, data compression and hierarchical multivariate curve resolution (H-MCR) were performed using custom scripts according to Trygg *et al*. (12). All manual ion integrations were performed using ChromaTOF 2.12 software (Leco Corp.) or custom scripts.

### Statistical analyses

The values are presented as median and range or means±s.e.m. For univariate data Student's *t*-test was used. All multivariate statistical analyses, i.e. principal component analysis (PCA) and orthogonal projections to latent structures-discriminant analysis (OPLS-DA), were performed using Simca software 11.0 (Umetrics, Umeå, Sweden). The following statistics for the OPLS-DA models are discussed in this article: R^2^X is the cumulative-modeled variation in X (metabolites); R^2^Y is the cumulative-modeled variation in Y (dummy variable for class discrimination); Q^2^Y is the cumulative-predicted variation in Y, according to cross-validation and p(corr) are the loadings scaled as correlation coefficients indicating correlation on class separation. The range of these parameters is 0–1, where 1 indicates a perfect fit. To present a manageable number of metabolites for further consideration, an arbitrary cutoff of |p(corr)|>0.7 was chosen for metabolites in this study. Bivariate correlations between variables were calculated using Pearson's correlation analysis. Statistical significance was set at *P*<0.05.

## Results

### Routine measurements

Baseline characteristics of the GHD patients are given in Table 1. The metabolic parameters for healthy controls and patients with GHD before and during GH replacement are summarised in Table 2. At baseline, IGF1 and HDL-cholesterol levels were lower in the patients with GHD than in the controls, whereas TG level BMI and waist circumference were higher. During GH replacement, IGF1 level increased with GH replacement (*P*=0.003), but no changes were seen in routine measurements.

### Metabolite profiles

**Figure 1:**
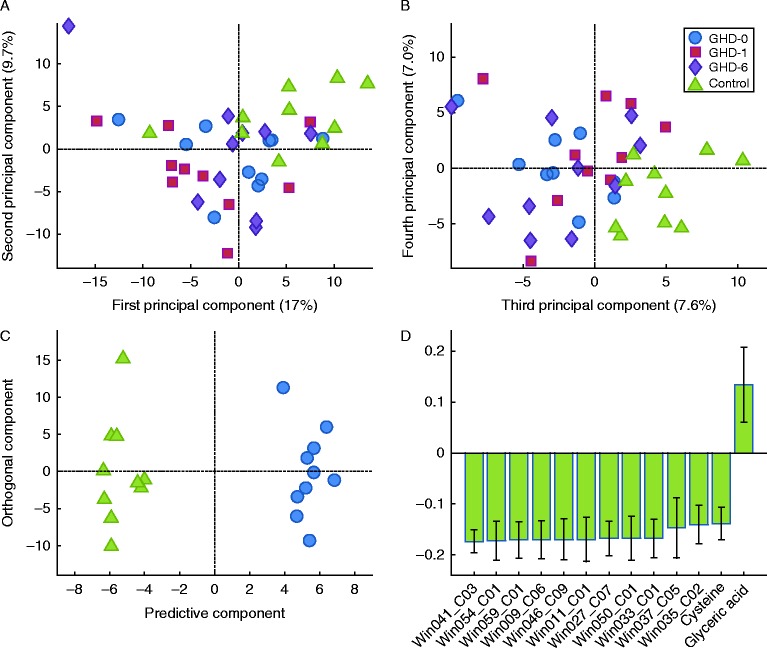
Multivariate analysis of metabolomic data obtained from serum samples of ten patients with growth hormone deficiency (GHD) during 6 month of GH replacement as compared with ten healthy controls. PCA score plots with the first and second principal component (A) and the third and fourth (B) indicate that the controls differ from GHD patients regardless of treatment. OPLS-DA score plots show the first predictive and orthogonal components (C) of a model with the healthy controls and the GHD patients at baseline. (D) Loadings (with a 95% CI) of metabolites with correlations coefficients above 0.70 are shown; the metabolites that have a high correlation with the separation between controls and patients. All these loadings were consistent over the three OPLS-DA models, except cysteine and glyceric acid, for which the correlations were 0.62 and 0.68 respectively in the comparison between healthy controls and GHD patients after 6 months of treatment. GHD-0, baseline; GHD-1, after 1 month of GH replacement; GHD-6, after 6 month of GH replacement.

To analyse metabolites from healthy controls and patients with GHD, the GC–MS data were processed using H-MCR, and 285 resolved components (putative metabolites) were obtained. The data were centred and scaled to unit variance before multivariate statistical projection methods, i.e. PCA and OPLS-DA (13). As shown in Fig. 1, by using the unsupervised PCA, a slight separation between healthy controls and GHD subjects was observed through the first four PCAs, but no trends or patterns could be observed in the samples of patients with GHD before and during GH replacement (Fig. 1A and B). To further evaluate the difference between healthy controls and GHD patients, and to investigate the difference between treated patients and untreated, supervised OPLS-DA was used. Dummy Y-vectors were created with 0 and 1 indicating one class or the other in pairwise models. The Y-vector was u.v.-scaled before analysis. The predictive models could not be built between treated and untreated GHD patients, but there were clear multivariate differences between GHD subjects and healthy controls (Fig. 1C). The multivariate statistics of the PCA and the OPLS-DA models are given in Table 3. Predictive OPLS-DA models were generated when healthy controls were compared with any patient group, but comparisons between the patient groups did not yield predictive models indicating that the multivariate metabolite difference in treatment of the GHD patients was not as pronounced. No further validation of the OPLS-DA models was conducted, except the cross-validation scheme within the model building process. Nonetheless, the three separate models with healthy controls and the three treatments groups were very similar, and as three different sets of samples were used and compared with healthy controls yielding resembling models, we conclude that there is a consistent multivariate metabolic difference between the serum samples of healthy controls and of GHD patients. In Fig. 1C, the OPLS-DA score plots of healthy controls vs GHD-patients at baseline are shown. The statistically significant loadings with a high correlation with class separation (|p(corr)|>0.7), in all three OPLS-DA models, are shown in Fig. 1D.

Among the 285 metabolites identified, 98 (34%) could be annotated. Thirteen metabolites were considered to be most important in separating GHD patients from healthy subjects, because of high correlation to class separation and consistency over all three OPLS-DA models (Fig. 1D). Eleven of them were unknown, but many could be classified as lipids, including phospholipids. Two were identified as cysteine and glyceric acid.

Using univariate analysis, 40 metabolites (14%) differed between healthy controls and patients with GHD (*P*<0.05), but none of these normalised upon GH treatment. Furthermore, 20 metabolites were significantly different after 1 month and 21 metabolites after 6 months of GH treatment. Seven metabolites were found to be altered in GHD patients, as well as affected by GH treatment, but only one (glyceric acid) could be annotated.

**Figure 2:**
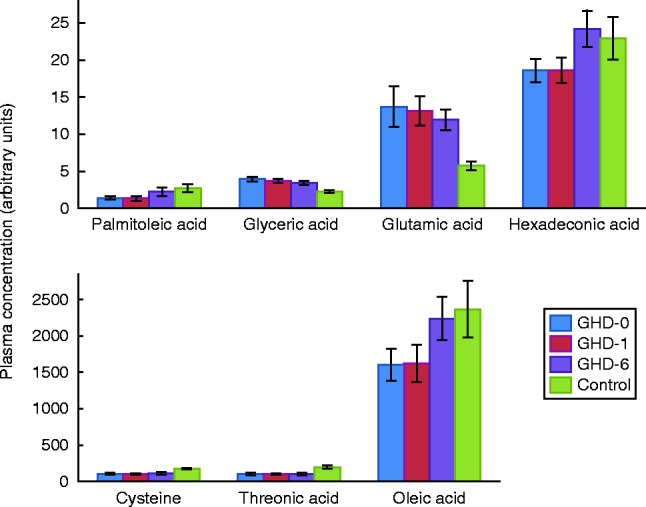
Effects of growth hormone (GH) on some of the known metabolites in the serum of ten patients with growth hormone deficiency (GHD) (*n*=10) and ten age and gender matched-controls (*n*=10) showing well-known effects of GH on amino acids and fatty acids. GHD-0, baseline; GHD-1, after 1 month of GH replacement; GHD-6, after 6 month of GH replacement.

Among the 98 annotated metabolites, nine were significantly different between GHD patients and controls. Threonic acid, cysteine, cystine and palmitoleic acid were found to be decreased in GHD patients, whereas glutamic acid, aspartic acid, hypoxanthine-like, uridine and glyceric acid were increased. A marked difference was observed in glyceric acid and glutamic acid. GH treatment tended to decrease the levels of these compounds towards the healthy control level (Fig. 2). In contrast, the levels of palmitoleic acid and hexadecanoic acid (C16:0) continuously increased during GH treatment (Fig. 2). Univariate statistical analysis (Student's *t*-test) identified seven annotated metabolites as being significantly different during GH treatment. Some incompletely identified compounds and guanosine decreased, whereas lysine, hexadecanoic acid (C16:0), oleic acid (C18:1), and one incompletely identified compound increased in GH treatment. The most pronounced effects were observed on fatty acid levels, which continuously changed towards the levels of the controls (Fig. 2).

The unique metabolic changes in LMC patterns were correlated with changes in IGF1 levels. The previously mentioned 40 compounds that differed between patients with GHD and healthy controls were singled out and showed increased or decreased levels in parallel with improved IGF1 levels. Individual levels of IGF1 were correlated with individual levels of LMCs. Cysteine (−0.62) and uridine (−0.62) correlated best with IGF1 levels in healthy subjects, whereas butanoic acid (0.61) and aspartic acid (0.65) correlated with IGF1 in patients with GHD before GH treatment was initiated. Glyceric acid (−0.67) correlated best with IGF1 levels after 1 month of GH treatment, and butanoic acid (0.71) and stearic acid (0.75) correlated with IGF1 levels after 6 months.

## Discussion

In this pilot study, we evaluated anthropometric and circulating metabolic parameters, including the metabolome, in ten adults with GHD before and after 1 and 6 months of GH replacement and compared the measurements with baseline measurements of ten age and gender matched-controls. Before GH treatment, BMI and waist circumference were higher in the patients with GHD. In addition, IGF1 and HDL-cholesterol levels were lower and TG levels higher in the GHD group. During 6 months of GH replacement, IGF1 increased, without changes in cholesterol, glucose, HbA1c, BMI or waist circumference. The analysis of the metabolome revealed a clearly different pattern in the patients with GHD, and most of the differing metabolites could be classified as lipids. During GH replacement, the well-known effects on amino and fatty acids were seen, but a majority of measured metabolites remained unchanged.

Relevant parameters from lipid and protein metabolism showed the expected changes during GH replacement. Detailed analysis of the metabolome showed that fatty acids were reduced in GHD patients and became normalised upon GH treatment, which is in accordance with the well-known lipolytic effect of GH (4, 5). Increased glutamic acid levels in GHD might be the result of a higher rate of transamination and protein degradation in muscle, fitting with the anabolic effects of GH (4, 5). The lower levels of cysteine and cystine in GHD could reflect an increased glutathione synthesis and may suggest higher oxidative stress in GHD patients. In the only previously published report on the metabolomics data of GHD patients (7) other amino acids were found to be changed. This could be caused by different age or different pituitary pathology and also indicated that more studies are needed to establish the use of metabolomics in patients with GHD. Glyceric acid can be obtained from the oxidation of glycerol and is a biomedical intermediate of lipid metabolism (14). It is interesting that GHD is characterised by an accumulation of glyceric acid and that an increase of this metabolite has previously been reported in obese children (15). However, the identified metabolites are of limited impact because the majority of metabolites of importance for the difference in patterns were not identified.

The so far unknown metabolites, which were altered the most in GHD and not normalised upon GH-treatment, might explain why the GH replacement did not reverse all the clinical symptoms of GHD, although IGF1 levels were normalised. Finding their identity might help to explain this problem.

The aim of this study was to evaluate whether the analysis of the metabolome would be a better marker in the diagnosis of GHD as well as in monitoring the effect of GH treatment. Our results showed a clear difference between GHD patients and healthy controls. Detailed analysis showed that the differing metabolites were mainly lipids and some of them were unknown. Analysis of the metabolome is complicated and more studies are needed to define the role of the metabolome in the diagnosis of GHD. We also evaluated the utility of a metabolomics technique to monitor the effects of GH treatment, and a combined analysis of around 250 metabolites showed only marginal changes during GH replacement. At the same time, IGF1 levels increased in all patients, and in this cohort analysis of metabolomics did not provide a better marker for treatment compared with IGF1.

It is assumed that disease-specific molecules will be leaked or be secreted into body fluids where they can be quantified (2). Factors such as age, gender, nutritional status and time of sampling might be reflected in the metabolite composition of the analysed body fluid. The heterogeneity of the GHD patients, which involves both the different pituitary diseases of the patients and the replacement therapy for the other pituitary deficiencies, pose a problem. Adults with GHD usually have several other pituitary insufficiencies, but a cohort of adults with isolated GHD would of course minimise the numbers of confounders. In an attempt to make the cohort more homogeneous, only patients with multiple insufficiencies on stable hormone replacements were included. The small number of studied individuals is also a potential limitation of these results.

In conclusion, the metabolome has unique features in GHD, but these metabolites were only marginally changed during GH treatment and no specific metabolite seemed to be a better marker than IGF1. The analysis of the metabolome is complicated and larger and longer studies are warranted to define its role in GHD.

## Figures and Tables

**Table 1:** Baseline characteristics of ten adults with growth hormone deficiency (GHD).

**Diagnosis**	**Gender** (M/F)	**Age** (years)	**Number of pituitary deficiencies in addition to GHD**	**IGF1 SDS**	**BMI** (kg/m^2^)	**Waist** (cm)
NFPA	F	26	4	−1.8	25.5	96
Ratke's cyst	F	34	3	−3.4	34.6	115
Cushing's disease	F	42	3	−3.7	20.5	80
Craniopharyngeoma	F	50	3	−4.1	25.6	89
Hypophysitis	F	57	3	−5.4	21.0	75
Prolactinoma	F	62	3	−1.9	24.4	86
NFPA	M	54	3	−1.7	28.0	98
Prolactinoma	M	56	3	−0.5	32.3	111
TBI	M	58	3	−1.3	27.5	111
Pituitary apoplexi	M	59	3	−0.7	28.2	100

IGF1, insulin-like growth factor 1; NFPA, non-functioning pituitary adenomas; TBI, traumatic brain injury.

**Table 2:** Metabolic and anthropometric characteristics (mean±s.d.) of ten healthy controls and ten patients with growth hormone (GH) deficiency at baseline and after 6 months.

	**Healthy controls**	**GHD baseline**	**GHD 6 months**
IGF1 (μg/l)	151±20	87±15*	172±37^†^
P-glucose (mmol/l)	5.1±0.2	4.6±0.3	4.8±0.4
HbA1c (%)	4.3±0.1	4.5±0.3	5.0±0.46
Total cholesterol (mmol/l)	5.3±0.4	5.9±0.4	ND
HDL-cholesterol (mmol/l)	1.8±0.2	1.4±0.2*	ND
LDL-cholesterol (mmol/l)	3.1±0.2	3.6±0.4	ND
Triglycerides (mmol/l)	0.9±0.2	1.9±0.3*	ND
BMI (kg/m^2^)	22±1	27±1*	27±1
Waist (cm)/hip (cm)	0.83±0.2	0.9±0.0*	0.9±0.0

IGF1, insulin-like growth factor 1, ND, not done. *P*<0.05: *between groups; ^†^within group.

**Table 3:** Statistics and characteristics of multivariate data analysis models.

**Model**	**Data**	**Number of samples in model**	**Number of variables in model**	**Model components** (orthogonal)	**R^2^X**	**R^2^Y**	**Q^2^Y**
OPLS	Healthy vs baseline	20	285+1	1 (1)	0.27	0.98	0.72
	Healthy vs 1 month	20	285+1	1 (1)	0.32	0.98	0.82
	Healthy vs 6 month	20	285+1	1 (1)	0.28	0.97	0.79
PCA	All samples	40	285	4	0.41		
